# P-1229. Assessment of Pharmacokinetic-Pharmacodynamic (PK-PD) Target Attainment for the Anti-Staphylococcal Antibiotic Afabicin

**DOI:** 10.1093/ofid/ofae631.1411

**Published:** 2025-01-29

**Authors:** David Cameron, Justine Dao, Christopher M Rubino, Annick Menetrey, Anthony P Cammarata, Jeffrey P Hammel, Guennaelle Dieppois, Justyna Nowakowska, Juan Bravo, Brian D VanScoy, Stephen Hawser, Valerie Nicolas-Metral, Sujata M Bhavnani

**Affiliations:** Debiopharm International, Lausanne, Vaud, Switzerland; Debiopharm International, Lausanne, Vaud, Switzerland; Institute for Clinical Pharmacodynamics, Schenectady, New York; Debiopharm International, Lausanne, Vaud, Switzerland; Institute for Clinical Pharmacodynamics, Schenectady, New York; Institute for Clinical Pharmacodynamics, Schenectady, New York; Debiopharm International, Lausanne, Vaud, Switzerland; Debiopharm International, Lausanne, Vaud, Switzerland; Debiopharm International, Lausanne, Vaud, Switzerland; Institute for Clinical Pharmacodynamics, Schenectady, New York; IHMA Europe, Monthey, Valais, Switzerland; Debiopharm International, Lausanne, Vaud, Switzerland; Institute for Clinical Pharmacodynamics, Schenectady, New York

## Abstract

**Background:**

Afabicin is a novel antimicrobial in clinical development for the treatment of staphylococcal infections. Its active moiety, afabicin desphosphono (Debio 1452), inhibits the essential enoyl-ACP reductase enzyme FabI, exerting antimicrobial effects by disrupting fatty acid biosynthesis. Herein, we describe an assessment of the probability of PK-PD target attainment (PTA) to support afabicin dose selection for patients with bone and joint infections (BJI).

**Figure:**
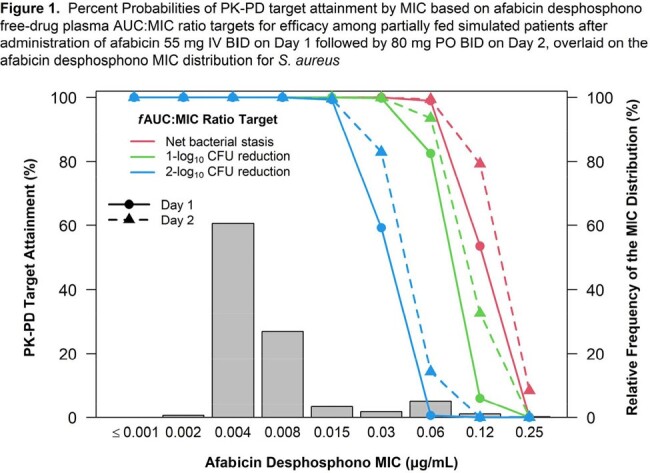

**Methods:**

MICs for 872 *Staphylococcus aureus* clinical isolates collected from 2017 to 2023 were determined according to CLSI guidelines (M07). Afabicin desphosphono plasma PK parameters from *S. aureus* infected mice were determined by non-compartmental analysis. Efficacy data for *S. aureus* (ATCC 33591, MIC = 0.015 µg/ml) were collected from a dose-ranging study conducted in an immunocompetent thigh infection model. The relationship between change in log_10_CFU from baseline and free-drug plasma AUC (*f*AUC):MIC ratio was evaluated by fitting a Hill-type model. A population PK model was developed using data from Phase 1 and an ongoing Phase 2 study in BJI. Monte Carlo simulations were used to generate *f*AUC in simulated patients in a partially fed state after administration of afabicin 55 mg IV BID on day 1 followed by 80 mg PO BID starting on day 2. PTA by MIC, and averaged over the MIC distribution was assessed on days 1 and 2.

**Results:**

Afabicin desphosphono displayed potent in vitro activity against *S. aureus* in vitro with a MIC90 of 0.015 µg/ml (range 0.002 – 0.25 µg/ml). MICs were similar for MSSA and MRSA. A Hill-type model described the PK-PD relationship in immunocompetent mice well. Based on this model, the derived fAUC:MIC ratio targets associated with net bacterial stasis, 1- and 2-log10CFU reductions were 2.2, 3.4, and 8.4, respectively. PTA on days 1 and 2 for the evaluated dosing regimen was ≥ 99.3% at the MIC90, and ≥ 92.7% averaged over the MIC distribution for *S. aureus* for all three targets (Figure 1).

**Conclusion:**

These data support the afabicin dosing regiment of 55 mg IV BID / 80 mg PO for the treatment of patients with *S. aureus* infections, which is further substantiated by clinical observations from an ongoing Phase 2 study in BJI (NCT03723551)

**Disclosures:**

**David Cameron, PhD**, Debiopharm International S.A.: Salary **Justine Dao, n/a**, Debiopharm International: Salary **Christopher M. Rubino, PharmD**, Achaogen Inc.: Grant/Research Support|Adagio Therapeutics, Inc.: Grant/Research Support|AiCuris Anti-infective Cures AG: Grant/Research Support|Albany Medical College: Grant/Research Support|AN2 Therapeutics: Grant/Research Support|Antabio SAS: Grant/Research Support|Apogee Biologics, Inc.: Grant/Research Support|Arcutis Biotherapeutics, Inc.: Grant/Research Support|B. Braun Medical Inc.: Grant/Research Support|Basilea Pharmaceutica: Grant/Research Support|BioFire Diagnostics, LLC.: Grant/Research Support|Cidara Therapeutics Inc.: Grant/Research Support|Cipla USA: Grant/Research Support|Cumberland Pharmaceuticals Inc.: Grant/Research Support|Entasis Therapeutics Inc., an affiliate of Innoviva Specialty Therapeutics, Inc.: Grant/Research Support|Excalibur Pharmaceuticals Inc.: Grant/Research Support|Fedora Pharmaceuticals: Grant/Research Support|Genentech: Grant/Research Support|GlaxoSmithKline: Grant/Research Support|Global Antibiotic Research and Development Partnership: Grant/Research Support|Hoffmann-La Roche: Grant/Research Support|Inotrem: Grant/Research Support|Insmed Inc.: Grant/Research Support|Institute for Clinical Pharmacodynamics, Inc.: Ownership Interest|Iterum Therapeutics Limited: Grant/Research Support|Kaizen Bioscience: Grant/Research Support|Lassen Therapeutics Inc.: Grant/Research Support|Matinas Biopharma: Grant/Research Support|Meiji Seika Pharma Co., Ltd.: Grant/Research Support|Melinta Therapeutics: Grant/Research Support|Mutabilis: Grant/Research Support|Nabriva Therapeutics AG: Grant/Research Support|Novobiotic Pharmaceuticals LLC.: Grant/Research Support|Paratek Pharmaceuticals, Inc.: Grant/Research Support|Pfizer Inc.: Grant/Research Support|Praxis Precision Medicines, Inc.: Grant/Research Support|PTC Therapeutics: Grant/Research Support|PureTech LYT 100 Inc.: Grant/Research Support|Qpex Biopharma: Grant/Research Support|Renibus Therapeutics: Grant/Research Support|Sfunga Therapeutics: Grant/Research Support|Shionogi Inc.: Grant/Research Support|Spero Therapeutics: Grant/Research Support|Spruce Biosciences Inc.: Grant/Research Support|Suzhou Sinovent Pharmaceuticals Co.: Grant/Research Support|Theravance: Grant/Research Support|University of Wisconsin: Grant/Research Support|US Food and Drug Administration: Grant/Research Support|UT Southwestern: Grant/Research Support|ValanBio therapeutics, Inc.: Grant/Research Support|VenatoRx: Grant/Research Support|Zogenix International: Grant/Research Support **Annick Menetrey, n/a**, Debiopharm International: Salary **Anthony P. Cammarata, M.S.**, Achaogen Inc.: Grant/Research Support|Adagio Therapeutics, Inc.: Grant/Research Support|AiCuris Anti-infective Cures AG: Grant/Research Support|Albany Medical College: Grant/Research Support|AN2 Therapeutics: Grant/Research Support|Antabio SAS: Grant/Research Support|Apogee Biologics, Inc.: Grant/Research Support|Arcutis Biotherapeutics, Inc.: Grant/Research Support|B. Braun Medical Inc.: Grant/Research Support|Basilea Pharmaceutica: Grant/Research Support|BioFire Diagnostics, LLC.: Grant/Research Support|Cidara Therapeutics Inc.: Grant/Research Support|Cipla USA: Grant/Research Support|Cumberland Pharmaceuticals Inc.: Grant/Research Support|Entasis Therapeutics Inc., an affiliate of Innoviva Specialty Therapeutics, Inc.: Grant/Research Support|Excalibur Pharmaceuticals Inc.: Grant/Research Support|Fedora Pharmaceuticals: Grant/Research Support|Genetech: Grant/Research Support|GlaxoSmithKline: Grant/Research Support|Global Antibiotic Research and Development Partnership: Grant/Research Support|Hoffmann-La Roche: Grant/Research Support|Inotrem: Grant/Research Support|Insmed Inc.: Grant/Research Support|Institute for Clinical Pharmacodynamics, Inc.: Employee|Iterum Therapeutics Limited: Grant/Research Support|Kaizen Bioscience: Grant/Research Support|Lassen Therapeutics Inc.: Grant/Research Support|Matinas Biopharma: Grant/Research Support|Meiji Seika Pharma Co., Ltd.: Grant/Research Support|Melinta Therapeutics: Grant/Research Support|Mutabilis: Grant/Research Support|Nabriva Therapeutics AG: Grant/Research Support|Novobiotic Pharmaceuticals LLC.: Grant/Research Support|Paratek Pharmaceuticals, Inc.: Grant/Research Support|Pfizer Inc.: Grant/Research Support|Praxis Precision Medicines, Inc.: Grant/Research Support|PTC Therapeutics: Grant/Research Support|PureTech LYT 100 Inc.: Grant/Research Support|Qpex Biopharma: Grant/Research Support|Renibus Therapeutics: Grant/Research Support|Sfunga Therapeutics: Grant/Research Support|Shionogi Inc.: Grant/Research Support|Spero Therapeutics: Grant/Research Support|Spruce Biosciences Inc.: Grant/Research Support|Suzhou Sinovent Pharmaceuticals Co.: Grant/Research Support|Theravance: Grant/Research Support|University of Wisconsin: Grant/Research Support|US Food and Drug Administration: Grant/Research Support|UT Southwestern: Grant/Research Support|ValanBio Therapeutics, Inc.: Grant/Research Support|VenatoRx: Grant/Research Support|Zogenix International: Grant/Research Support **Jeffrey P. Hammel, MS**, Achaogen Inc.: Grant/Research Support|Adagio Therapeutics, Inc.: Grant/Research Support|AiCuris Anti-infective Cures AG: Grant/Research Support|Albany Medical College: Grant/Research Support|AN2 Therapeutics: Grant/Research Support|Antabio SAS: Grant/Research Support|Apogee Biologics, Inc.: Grant/Research Support|Arcutis Biotherapeutics, Inc.: Grant/Research Support|B. Braun Medical Inc.: Grant/Research Support|Basilea Pharmaceutica: Grant/Research Support|BioFire Diagnostics, LLC.: Grant/Research Support|Cidara Therapeutics Inc.: Grant/Research Support|Cipla USA: Grant/Research Support|Cumberland Pharmaceuticals Inc.: Grant/Research Support|Entasis Therapeutics Inc., an affiliate of Innoviva Specialty Therapeutics, Inc.: Grant/Research Support|Excalibur Pharmaceuticals Inc.: Grant/Research Support|Fedora Pharmaceuticals: Grant/Research Support|Genetech: Grant/Research Support|GlaxoSmithKline: Grant/Research Support|Global Antibiotic Research and Development Partnership: Grant/Research Support|Hoffmann-La Roche: Grant/Research Support|Inotrem: Grant/Research Support|Insmed Inc.: Grant/Research Support|Institute for Clinical Pharmacodynamics, Inc.: Employee|Iterum Therapeutics Limited: Grant/Research Support|Kaizen Bioscience: Grant/Research Support|Lassen Therapeutics Inc.: Grant/Research Support|Matinas Biopharma: Grant/Research Support|Meiji Seika Pharma Co., Ltd.: Grant/Research Support|Melinta Therapeutics: Grant/Research Support|Mutabilis: Grant/Research Support|Nabriva Therapeutics AG: Grant/Research Support|Novobiotic Pharmaceuticals LLC.: Grant/Research Support|Paratek Pharmaceuticals, Inc.: Grant/Research Support|Pfizer Inc.: Grant/Research Support|Praxis Precision Medicines, Inc.: Grant/Research Support|PTC Therapeutics: Grant/Research Support|PureTech LYT 100 Inc.: Grant/Research Support|Qpex Biopharma: Grant/Research Support|Renibus Therapeutics: Grant/Research Support|Sfunga Therapeutics: Grant/Research Support|Shionogi Inc.: Grant/Research Support|Spero Therapeutics: Grant/Research Support|Spruce Biosciences Inc.: Grant/Research Support|Suzhou Sinovent Pharmaceuticals Co.: Grant/Research Support|Theravance: Grant/Research Support|University of Wisconsin: Grant/Research Support|US Food and Drug Administration: Grant/Research Support|UT Southwestern: Grant/Research Support|ValanBio Therapeutics, Inc.: Grant/Research Support|VenatoRx: Grant/Research Support|Zogenix International: Grant/Research Support **Guennaelle Dieppois, n/a**, Debiopharm International: Salary **Justyna Nowakowska, n/a**, Debiopharm International: Salary **Juan Bravo, n/a**, Debiopharm International: Salary **Brian D. VanScoy, B.S.**, Achaogen Inc.: Grant/Research Support|Adagio Therapeutics, Inc.: Grant/Research Support|AiCurtis Anti-infective Cures AG: Grant/Research Support|Albany Medical College: Grant/Research Support|AN2 Therapeutics: Grant/Research Support|Antabio SAS: Grant/Research Support|Apogee Biologics, Inc: Grant/Research Support|Arcutis Biotherapeutics, Inc.: Grant/Research Support|B. Braun Medical Inc.: Grant/Research Support|Basilea Pharmaceutica: Grant/Research Support|BioFire Diagnostics, LLC.: Grant/Research Support|Cidara Therapeutics Inc.: Grant/Research Support|Cipla USA: Grant/Research Support|Cumberland Pharmaceuticals Inc.: Grant/Research Support|Entasis Therapeutics: Grant/Research Support|Excalibur Pharmaceuticals Inc.: Grant/Research Support|Fedora Pharmaceuticals: Grant/Research Support|Genentech: Grant/Research Support|GlaxoSmithKline: Grant/Research Support|Global Antibiotic Research and Development Partnership: Grant/Research Support|Hoffmann-La Roche: Grant/Research Support|ICPD: Employee|Inotrem: Grant/Research Support|Insmed Inc: Grant/Research Support|Iterum Therapeutics Limited: Grant/Research Support|Kaizen Bioscience: Grant/Research Support|Lassen Therapeutics Inc.: Grant/Research Support|Matinas Biopharma: Grant/Research Support|Meiji Seika Pharma Co., Ltd.: Grant/Research Support|Melinta Therapeutics: Grant/Research Support|Mutabilis: Grant/Research Support|Nabriva Therapeutics AG: Grant/Research Support|Novobiotic Pharmaceuticals LLC: Grant/Research Support|Paratek Pharmaceuticals, Inc.: Grant/Research Support|Pfizer Inc: Grant/Research Support|Praxis Precision Medicines, Inc.: Grant/Research Support|PTC Therapeutics: Grant/Research Support|PureTech LYT 100 Inc.: Grant/Research Support|Qpex Biopharma: Grant/Research Support|Renibus Therapeutics: Grant/Research Support|Sfunga Therapeutics: Grant/Research Support|Shionogi Inc.: Grant/Research Support|Spero Therapeutics: Grant/Research Support|Spruce Biosciences Inc.: Grant/Research Support|Suzhou Sinovent Pharmaceuticals Co: Grant/Research Support|Theravance: Grant/Research Support|University of Wisconsin: Grant/Research Support|US Food and Drug Administration: Grant/Research Support|UT Southwestern: Grant/Research Support|ValanBio Therapeutics, Inc.: Grant/Research Support|VenatoRx: Grant/Research Support|Zogenix International: Grant/Research Support **Valerie Nicolas-Metral, PhD**, Debiopharm International: Salary **Sujata M. Bhavnani, PharmD; MS; FIDSA**, Achaogen Inc.: Grant/Research Support|Adagio Therapeutics, Inc.: Grant/Research Support|AiCuris Anti-infective Cures AG: Grant/Research Support|Albany Medical College: Grant/Research Support|AN2 Therapeutics: Grant/Research Support|Antabio SAS: Grant/Research Support|Apogee Biologics, Inc.: Grant/Research Support|Arcutis Biotherapeutics, Inc.: Grant/Research Support|B. Braun Medical Inc.: Grant/Research Support|Basilea Pharmaceutica: Grant/Research Support|BioFire Diagnostics, LLC.: Grant/Research Support|Cidara Therapeutics Inc.: Grant/Research Support|Cipla USA: Grant/Research Support|Cumberland Pharmaceuticals Inc.: Grant/Research Support|Entasis Therapeutics Inc., an affiliate of Innoviva Specialty Therapeutics, Inc.: Grant/Research Support|Excalibur Pharmaceuticals Inc.: Grant/Research Support|Fedora Pharmaceuticals: Grant/Research Support|Genetech: Grant/Research Support|GlaxoSmithKline: Advisor/Consultant|GlaxoSmithKline: Grant/Research Support|Global Antibiotic Research and Development Partnership: Grant/Research Support|Hoffmann-La Roche: Grant/Research Support|Inoterm: Grant/Research Support|Insmed Inc.: Grant/Research Support|Institute for Clinical Pharmacodynamics, Inc.: Ownership Interest|Iterum Therapeutics Limited: Grant/Research Support|Kaizen Bioscience: Grant/Research Support|Lassen Therapeutics Inc.: Grant/Research Support|Matinas Biopharma: Grant/Research Support|Meiji Seika Pharma Co., Ltd.: Grant/Research Support|Melinta Therapeutics: Grant/Research Support|Mutabilis: Grant/Research Support|Nabriva Therapeutics AG: Grant/Research Support|Novobiotic Pharmaceuticals LLC.: Grant/Research Support|Paratek Pharmaceuticals, Inc.: Grant/Research Support|Pfizer Inc.: Grant/Research Support|Praxis Precision Medicines, Inc.: Grant/Research Support|PTC Therapeutics: Grant/Research Support|PureTech LYT 100 Inc.: Grant/Research Support|Qpex Biopharma: Grant/Research Support|Renibus Therapeutics: Grant/Research Support|Sfunga Therapeutics: Grant/Research Support|Shionogi Inc.: Advisor/Consultant|Shionogi Inc.: Grant/Research Support|Spero Therapeutics: Grant/Research Support|Spruce Biosciences Inc.: Grant/Research Support|Suzhou Sinovent Pharmaceuticals Co.: Grant/Research Support|Theravance: Grant/Research Support|University of Wisconsin: Grant/Research Support|US Food and Drug Administration: Grant/Research Support|UT Southwestern: Grant/Research Support|ValanBio Therapeutics, Inc.: Grant/Research Support|VenatoRx: Grant/Research Support|Zogenix International: Grant/Research Support

